# A New Sesquiterpene Essential Oil from the Native Andean Species *Jungia rugosa* Less (Asteraceae): Chemical Analysis, Enantiomeric Evaluation, and Cholinergic Activity

**DOI:** 10.3390/plants10102102

**Published:** 2021-10-04

**Authors:** Karyna Calvopiña, Omar Malagón, Francesca Capetti, Barbara Sgorbini, Verónica Verdugo, Gianluca Gilardoni

**Affiliations:** 1 Departamento de Química, Universidad Técnica Particular de Loja, Calle M. Champagnat s/n, Loja 110107, Ecuador; kmcalvopina1@utpl.edu.ec (K.C.); omalagon@utpl.edu.ec (O.M.); vmverdugo@utpl.edu.ec (V.V.); 2 Carrera de Ingeniería Química, Facultad de Ingenierías, Universidad Técnica “Luis Vargas Torres” de Esmeraldas, Ciudadela Nuevos Horizontes s/n, Esmeraldas 179619, Ecuador; 3 Dipartimento di Scienza e Tecnologia del Farmaco, Università degli Studi di Torino, 10125 Torino, Italy; francesca.capetti@unito.it (F.C.); barbara.sgorbini@unito.it (B.S.); 4 Unidad Educativa Ambrosio Andrade Palacios-Suscal, Vía Durán Tambo Eloy Alfaro, Suscal 030206, Ecuador

**Keywords:** *Jungia rugosa*, *Jungia bullata*, *Jungia jelskii*, *Jungia malvifolia*, Asteraceae, essential oil, enantiomers, sesquiterpenes, Ecuador

## Abstract

As part of a project devoted to the phytochemical study of Ecuadorian biodiversity, new essential oils are systematically distilled and analysed. In the present work, *Jungia rugosa* Less (Asteraceae) has been selected and some wild specimens collected to investigate the volatile fraction. The essential oil, obtained from fresh leaves, was analysed for the first time in the present study. The chemical composition was determined by gas chromatography, coupled to mass spectrometry (GC-MS) for qualitative analysis, and to flame ionization detector (GC-FID) for quantitation. The calculation of relative response factors (RRF), based on combustion enthalpy, was carried out for each quantified component. Fifty-six compounds were identified and quantified in a 5% phenyl-polydimethylsiloxane non-polar column and 53 compounds in a polyethylene glycol polar column, including four undetermined compounds. The main feature of this essential oil was the exclusive sesquiterpenes content, both hydrocarbons (74.7% and 80.4%) and oxygenated (8.3% and 9.6%). Major constituents were: γ-curcumene (47.1% and 49.7%) and β-sesquiphellandrene (17.0% and 17.9%), together with two abundant undetermined oxygenated sesquiterpenes, whose abundance was 6.7–7.2% and 4.7–3.3%, respectively. In addition, the essential oil was submitted to enantioselective evaluation in two β-cyclodextrin-based enantioselective columns, determining the enantiomeric purity of a minor component (1*S*,2*R*,6*R*,7*R*,8*R*)-(+)-α-copaene. Finally, the AChE inhibition activity of the EO was evaluated in vitro. In conclusion, this volatile fraction is suitable for further investigation, according to two main lines: (a) the purification and structure elucidation of the major undetermined compounds, (b) a bio-guided fractionation, intended to investigate the presence of new sesquiterpene AChE inhibitors among the minor components.

## 1. Introduction

Ecuador, due to multiple combinations of factors, has been configured as a megadiverse country, with a high rate of plant endemism per surface area, which makes it one of the richest countries in biodiversity and endemism of the world [1,2]. Some figures presented in the Fifth and Sixth National Report for the Convention on Biological Diversity regarding the emergence of new plant species illustrate this peculiarity: between 1999 and 2012, 2443 new species were reported for the country, of which 1663 were also new to the science. In 2013, 18,198 species of vascular plants were registered, which meant 1140 more species than those reported in 2010 and representing about 7.6% of the vascular plants registered worldwide. It is estimated that the total number of vascular plants could reach 25,000 [3,4].

Along with the above, indigenous cultures possess a strong tradition about plants as a means of treating diseases, which has allowed ancestral knowledge to be transferred through generations from ancient times to the present, promoting the abundant use of medicinal plants. For all these reasons, Ecuador is an invaluable source of natural products and unprecedented knowledge about plant applications. In contrast, the number of high-impact scientific studies in this area is relatively low, given the potential that the country’s biodiversity offers [5]. In this respect, to the best of the authors’ knowledge, the essential oil (EO), distilled from the leaves of *Jungia rugosa* Less, has never been described.

Within the Asteraceae, the *Jungia* genus corresponds to flowering plants that mostly develop at high altitudes and cold climates, being characteristic of the Andean regions of Ecuador, Peru, and Argentina. Despite many articles describing the phytochemistry of genus *Jungia*, only three deal with EOs. In fact, only the volatile fractions of *Jungia paniculata* and *Jungia polita* have been described so far, the first one being very popular in the Andes and known with the traditional name “matico” [6,7,8]. Concerning *J. rugosa*, two phytochemical studies have been published. However, they are devoted to non-volatile fractions and their biological activities [9,10].

*Jungia rugosa* Less (Asteraceae) is a native Andean species, growing at altitudes between 1500 and 4000 m above sea level [11]. It is characterised by great resistance to frost and low temperatures, which is why it prevails in cold and humid climates. This plant grows up to 5 m in height, presenting a thin, woody, smooth, hard, and green stem. Its intense green leaves with a pale green underside, measure between 5 and 12 centimetres and are covered with villi; they are also petiolate, presenting an anti-parallel rib. Its main root divides, giving rise to an abundant root system. Its flower is whitish in colour, presented in a green capsule, which generates small black seeds. In some localities located in the Andean region of Ecuador, it is better known as “carne humana”. Based on the indigenous heritage of the central Ecuadorian region (Cotopaxi), this species is used as an anti-inflammatory remedy, for instance, in treating bruises, and for other unspecified healing purposes [12]. The anti-inflammatory activity is probably the most important medicinal property of this plant, since it has also been confirmed by two scientific studies, together with the closely related antioxidant capacity [13,14]. Some sources also report that leaf decoctions are applied to treat wounds and skin ulcerations, gastric problems, and kidney disorders, among others [15,16]. In addition to medicinal applications, this species is also used to prepare ropes in the Chimborazo region of Ecuador [12]. Furthermore, *J. rugosa* is also known with three botanical synonyms: *Jungia bullata* Turcz., *Jungia jelskii* Hieron., and *Jungia malvifolia* Muschl [17]. None of these synonyms corresponds to any chemical literature.

So far, many plant species from Ecuador have been described for producing new EOs, often characterised by important biological activities such as analgesic, antioxidant, antibacterial, anticancer, and sedative, among others [5,18,19,20]. In particular, EOs rich in sesquiterpenes have been presented as promising anti-proliferative agents, whose constituents are able to easily reach certain organs, such as heart, liver, and kidneys [20]. Among all the biological properties of EOs and their constituents, we are particularly interested in the inhibition of acetylcholinesterase (AChE) and butyrylcholinesterase (BChE), due to the serious implications that neurodegenerative diseases are ever-more producing in western countries [18,21,22,23].

In accordance with the above, the objectives of this research were to investigate the chemical and enantiomeric composition of *J. rugosa* EO and to evaluate the presence of cholinergic molecules in this volatile fraction. All this information will provide a contribution to the phytochemical and phytopharmacological knowledge of the Ecuadorian flora.

## 2. Results

### 2.1. Distillation and Physical Properties

The essential oil of the fresh aerial parts of *Jungia rugosa* was obtained by steam distillation for 4 h, yielding an average of 0.09 (*w*/*w*). The physical properties, chemical composition and enantiomeric analysis are discussed below.

Two physical properties were determined: relative density (*d* = 0.898 ± 0.012 g/mL) and refractive index (*η* = 1.505 ± 0.002). These properties are notoriously determined by genetic characteristics, geographical location, and phenological stage of the plant [24].

### 2.2. Chemical Analysis of the EO

In the chemical analysis of the EO of *J. rugosa*, all components identified were sesquiterpenes corresponding to a total of 56 and 53 compounds, respectively, with DB-5ms and HP-INNOWax columns (see Section 4.4). Most of the constituents (52) were identified by comparing the electron impact mass spectrum (EIMS) and the linear retention index (LRI) with literature, whereas four remained unidentified. According to their molecular weight, the unknown components are consistent with one sesquiterpene (204 amu) and three oxygenated sesquiterpenoids (220, 262 and 280 amu). The significant difference between calculated and reference LRIs is within the experimental error.

**Figure 1:**
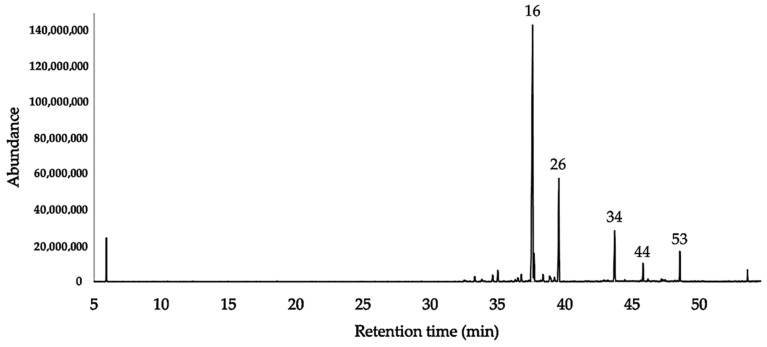
GC-MS chromatogram of *J. rugosa* EO on DB-5ms column.

**Figure 2:**
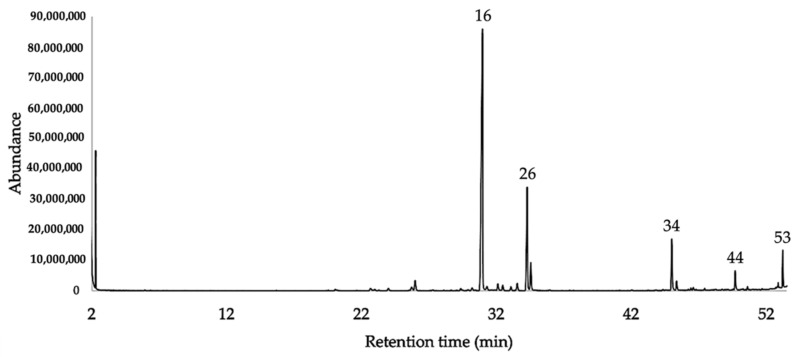
GC-MS chromatogram of *J. rugosa* EO on HP-INNOWax column.

Concerning the quantitative analysis (see Section 4.5), 50 compounds were quantified on at least one column, with a detection threshold of 0.1%, whereas six compounds (β-cubebene, α-chamipinene, δ-amorphene, *allo*-aromadendrene epoxide, *cis*-thujopsenal, and 8-α-acetoxyelemol) appeared as traces (<0.1%) in both columns. Quantified components corresponded to 98.3% and 99.6% of the EO total mass, on the non-polar and polar column, respectively; the sesquiterpene hydrocarbons, corresponding to 75.8% and 80.8% and oxygenated sesquiterpenes, corresponding to 22.3–18.8%, respectively. The major components, with an average amount ≥3% over the two columns, were γ-curcumene (47.1%, 49.7%), β-sesquiphellandrene (17.0%, 17.9%), *ar*-curcumene (3.4%, 4.2%) and two undetermined oxygenated sesquiterpenes with molecular weight 220 (6.7%, 7.2%) and 262 (4.7%, 3.3%). A standard deviation of less than 5% was obtained between the percentages of each analyte with both columns. The GC-MS chromatograms on both columns are reported in Figure 1 and Figure 2. Table 1 shows the identified components together with their relative percent abundance, calculated vs. *n*-nonane as the internal standard.

### 2.3. Enantioselective Evaluation of the EO

The enantioselective analysis of the EO was carried out on a 2,3-diethyl-6-*tert*-butyldimethylsilyl-β-cyclodextrin based capillary column. Only the very minor compound (1*S*,2*R*,6*R*,7*R*,8*R*)-(+)-*α*-copaene could be certainly identified, appearing enantiomerically pure in the EO. No more enantiomeric pairs or enantiomerically pure compounds could be identified in the enantioselective chromatogram.

### 2.4. AChE Inhibition Activity

The AChE inhibitory activity of the investigated essential oils was measured in vitro, using a spectrophotometric assay based on Elman’s method. Galanthamine and *Laurus nobilis* EO were used as positive controls, the latter being considered an active EO in literature (see Section 3. Discussion). All results are summarised in Table 2.

## 3. Discussion

### 3.1. The Chemical Composition

About the chemical composition of the EO, the hydrocarbon sesquiterpene fraction was predominant, corresponding to 74.7% and 80.4% with a non-polar and a polar column respectively. Furthermore, an oxygenated sesquiterpene fraction was present between 9.6% and 8.3% of the whole amount. No monoterpenes were detected in the EO. Major components of this volatile fraction were γ-curcumene and β-sesquiphellandrene, together with two undetermined oxygenated sesquiterpenes (molecular weight 220 amu and 262 amu, respectively). If we compare these results with the only two partial analyses, known so far for EOs of genus *Jungia* (*J. paniculata* and *J. polita*), the prevalence of sesquiterpenes is confirmed [7,8]. However, unlike our case, (*E*)-β-caryophyllene and caryophyllene oxide are there the main components. Regarding γ-curcumene, it derives its name from *Curcuma longa* L. (turmeric), but we must look at *Helichrysum italicum* (Roth) G. Don (Asteraceae) to find an important and widely studied botanical species where γ-curcumene is often a major constituent. Other *Helichrysum* species are also familiar with similar sesquiterpene compositions [49]. On the one hand, despite γ-curcumene being quite common and known for a long time, no exhaustive studies on its pharmacology can be found. On the other hand, the EOs where it is an important component are widely described, with all the typical biological activities known for volatile fractions. In regards to β-sesquiphellandrene, it is also a typical hydrocarbon sesquiterpene of *Curcuma longa*. The most important study on its pharmacological properties is probably a recent publication, where β-sesquiphellandrene has been described as a potent anticancer agent. Its activity is comparable with the one of curcumin. According to that investigation, β-sesquiphellandrene would exert an antiproliferative activity, by inhibiting the formation of cancer cell colonies and inducing apoptosis. The neoplastic formations, that appeared to be more sensitive to this metabolite, were leukaemia, multiple myeloma, and colorectal cancer. Furthermore, cancer cells expressing p-53 protein resulted in being more sensitive to β-sesquiphellandrene than those lacking it [50]. Finally, we must mention the presence of two important undetermined compounds, contributing to the mass of the EO with the non-negligible amounts of 6.7–7.2% and 4.7–3.3%, respectively (see peaks 34 and 53 in Figure 1 and Figure 2). These constituents showed a molecular ion of 220 and 262 amu—the first one being characteristic of sesquiterpenoids with molecular formula C_15_H_24_O, whereas the second one is consistent with the rare sesquiterpene derivatives of formula C_18_H_30_O (e.g., sesquiterpenes acetones) [25].

### 3.2. The Enantiomeric Evaluation

For what concerns the enantiomeric evaluation, the EO was submitted to enantioselective GC, in a classical β-cyclodextrin-based capillary column. However, the only chiral terpene that could be identified was (1*S*,2*R*,6*R*,7*R*,8*R*)-(+)-*α*-copaene, present as a pure enantiomer. No other sesquiterpene could be detected, both as a pure enantiomer or enantiomeric pair. This result is not surprising. In fact, most of the enantiomerically pure available standards are indeed monoterpenes, whose use is necessary to determine the elution order of the enantiomers from an enantioselective column. Since the present EO is exclusively constituted by sesquiterpenes, the corresponding enantioselective GC information is, for most of them, unavailable. Furthermore, the similarity among the spectra for this class of metabolites excluded the possibility to certainly identify enantiomeric pairs within the peaks. The only exception, although a minor component, was (1*S*,2*R*,6*R*,7*R*,8*R*)-(+)-*α*-copaene, since the enantiomerically pure standards of this compound are available.

### 3.3. The Cholinergic Activity

Finally, the inhibition activity of this EO against AChE can be discussed. Observing our results, shown in Table 2, the inhibition capacity of *J. rugosa* EO was compared to the ones of galanthamine and *L. nobilis* EO. However, whereas the biological activity of galanthamine is clearly extremely high, mainly because it is a pure compound, the biological activity of *L. nobilis* EO is decidedly lower. Nevertheless, *L. nobilis* EO is considered as an active mixture in this kind of assay, and it can be subsequently used as a better positive control while working with EOs [51]. In our case, the inhibition power of *J. rugosa* EO is about 68% compared to that of *L. nobilis* EO, clearly resulting in less activity but not inactive. This fact could be explained by the presence of at least one active minor sesquiterpene in the mixture. If that is the case, the EO may be considered useless as it is, but suitable to be studied, through a bio-guided fractionation, in search of new sesquiterpene inhibitors. The interest in this aspect resides in that, to the best of the authors’ knowledge, the most active EOs are characterised by an important monoterpene fraction (except for the case where the EO is dominated by (*E*)-β-caryophyllene) [18,22]. However, due to their toxicity, hydrocarbon monoterpenes can hardly be used as pharmaceutical active principles, which cannot be assumed for sesquiterpenes. Therefore, the discovery of new sesquiterpene inhibitors of AChE is a matter of some pharmaceutical interest. Consequently, this volatile fraction is suitable for further investigation, according to two main lines: a) the purification and structure elucidation of the major undetermined compounds, by mean of preparative chromatography and NMR spectroscopy; b) a bio-guided preparative fractionation, intended to investigate the presence of new sesquiterpene AChE inhibitors among the minor components. Due to the low distillation yield of this EO, a non-classical approach should be applied. On the one hand, a tentative method for purification and structure elucidation could be the use of preparative thin-layer chromatography (TLC) and micro-probe NMR spectroscopy. In this way, about 1 mg of a pure compound would be enough to be submitted to a complete series of NMR experiments. On the other hand, the bio-guided investigation could be faced through a bioautographic method. Based on a TLC analysis, a bioautographic assay can be carried out with few micrograms of EO. Since the active compounds possibly are known sesquiterpenes, the combined use of bioautography, preparative TLC and GC-MS should afford the desired information.

In regards to the traditional use of *J. rugosa*, some previous studies have described the antioxidant and anti-inflammatory activities of the non-volatile fraction, mainly attributed to flavonoids [13,14]. Since these properties are fully consistent with the ethnobotanical use, the EO could probably be exempted to be considered the active fraction.

## 4. Materials and Methods

### 4.1. General Information

The chemical and enantioselective analyses of the *J. rugosa* EO were performed with a gas chromatography-mass spectrometry (GC-MS) system, consisting of a 6890 N Agilent Technologies gas chromatograph with an autoinjector model 7683. The instrument was coupled to an Agilent Technologies simple quadrupole mass spectrometry detector (MSD) model 5973 INERT (Santa Clara, CA, USA), and a common flame ionization detector (FID). The MSD operated in SCAN mode (scan range 35–350 *m*/*z*), with an electron ionization (EI) source at 70 eV. The qualitative and quantitative analyses were carried out with both non-polar and polar stationary phase capillary columns from Agilent Technologies. The non-polar column was based on 5% phenyl-methylpolysiloxane phase (DB-5ms, 30 m long, 0.25 mm internal diameter, and 0.25 μm film thickness), while the polar column was provided with a polyethylene glycol stationary phase (HP-INNOWax, 30 m × 0.25 mm × 0.25 μm). The enantioselective analysis was run with an enantioselective capillary column, based on 30% diethyl-*tert*-butyldimethylsilyl-*β*-cyclodextrin in PS-086 as chiral stationary phases as a chiral selector (25 m × 250 µm internal diameter × 0.25 µm phase thickness, purchased from Mega, MI, Italy). For all the analyses, GC purity grade helium (Indura, Guayaquil, Ecuador) was used as the carrier gas, set at the constant flow, with a rate of 1 mL/min. For the biological assays, a Spectronic Genesys 6 spectrophotometer was used, purchased from Thermo-Fisher Scientific (Waltham, MA, USA).

All solvents for GC analysis, the mixture of *n*-alkanes C_9_–C_25_ for linear retention indices (LRI), internal standard (*n*-nonane), and reagents for the inhibition activity assays were purchased from Sigma-Aldrich. The calibration standard was isopropyl caproate, obtained by synthesis in the authors’ laboratory and purified to 98.8% (GC-FID).

### 4.2. Plant Material

The leaves of *J. rugosa* were collected in February 2020 in the south-central area of the Ecuadorian highlands (sector Citar, province Cañar). The specimens grew at an altitude of 3445 m above sea level, with coordinates 02°35′387″ S and 78°56′309″ W. The collection was carried out under governmental permission (MAAE-ARSFC-2020-0638), issued by the Ministry of Environment of Ecuador (MAE). The identification was achieved at the Universidad Técnica Particular de Loja, by the botanist Dr. Itziar Arnelas, and a botanical specimen was deposited at the UTPL herbarium, with voucher number D-HUTPL-2020-6. The fresh leaves of *J. rugosa* were distilled the day after collection.

### 4.3. Distillation of the EO and GC Sample Preparation

Fresh leaves (4.8 kg) of *J. rugosa* were steam distilled in a Clevenger-type apparatus for 4 h. The EO, which spontaneously separated from the aqueous layer, was immediately dried on anhydrous sodium sulphate and stored in the dark at −15 °C until use. The analytical samples for each GC injection were prepared as described in previous studies [18,19,20,21,22].

### 4.4. GC-MS Qualitative Analyses

The EO was analysed by injecting 1 µL of analytical sample into the GC instrument that operated in split mode (40:1). The injector temperature was set at 220 °C. The elution with the DB-5ms column was conducted according to the following oven temperature program: 60 °C was kept for 5 min, followed by a first thermal gradient to 100 °C at a rate of 2 °C/min, a second gradient to 150 °C at a rate of 3 °C/min, then a third gradient to 200 °C at a rate of 5 °C/min; in the end, the oven temperature was maintained at 250 °C for 15 min at a rate of 15 °C/min. With the HP-INNOWax column, the same conditions and oven program were applied, except for the final temperature, which was set at 230 °C.

In order to identify the components of the EO, a homologous series of *n*-alkanes, from *n*-nonane to *n*-pentacosane, was injected in each column. The linear retention index (LRI) of each constituent was calculated according to Van Den Dool and Kratz [48]. This way each volatile metabolite was identified by comparing the corresponding LRI value and EI-MS spectrum with tabulated data for DB-5ms [25] and HP-INNOWax [26,27,28,29,30,31,32,33,34,35,36,37,38,39,40,41,42,43,44,45,46,47].

### 4.5. GC-FID Quantitative Analyses

The quantitative analyses were performed, with both columns, under the same conditions and configurations described for the qualitative ones. All the samples were injected in quadrupled and the percentage of each analyte in the EO was calculated as the average value over the four injections. The quantification was achieved by external calibration and use a process internal standard, calculating the relative response factor (RRF) of each EO constituent, based on its combustion enthalpy [52]. The original method was modified, isopropyl caproate instead of the methyl octanoate reported in the literature, was chosen as the calibration standard for this analysis. This approach is based on the principle that the RRF of an organic compound only depends on the molecular formula and number of aromatic rings, being the same for isomers.

Two external calibration curves were build-up, according to what is described in previous articles [18,19,20,21,22]. All calibration curves achieved an R^2^ > 0.995.

### 4.6. Enantioselective Analysis of the EO

The enantioselective analysis of the EO was carried out by GC-MS on the same samples of the qualitative and quantitative analyses. The injector temperature was the same as for the EO qualitative analysis, whereas the injector operated in split mode, with a ratio of 50:1. The following oven thermal program was applied: The initial temperature was 60 °C for 2 min, followed by a thermal gradient of 2 °C/min until 220 °C, maintained for 2 min. In addition, a mixture of *n*-alkanes (C_9_–C_25_) was injected under the same conditions as for conventional analysis to determine LRIs. The enantiomeric pairs of chiral sesquiterpenes were identified based on the EI-MS spectra and elution order, determined according to literature data for the same chiral selector [53,54].

### 4.7. AChE Inhibition Spectrophotometric Assay

The protocol followed in this study was that of Rhee et al., with slight modifications [55]. Enzyme solution 0.22 U/mL was prepared in 50 mM Tris-HCl, pH 8, containing 0.1% bovine serum albumin (BSA). Acetyltiocholine (ATCI) solution 1.5 mM was prepared in Millipore water. Ellman’s reagent solution 3mM was prepared in 50 mM Tris–HCl, pH 8, containing 0.1 M NaCl and 0.02 M MgCl_2_ hexahydrate. Essential oils stock solutions 15 mg/mL and 30 mg/mL were prepared in dimethyl sulfoxide (DMSO). *Laurus nobilis* essential oil solution 15 mg/mL and galanthamine solution 0.4 mg/mL in DMSO were used as a positive control.

The reagents were placed in the cuvette in the following order: 150 µL of ATCI solution, 900 µL of buffer B, 5 µL of the essential oil/galanthamine solution and finally 150 µL of enzyme solution. BSA 1%, Tween-20 0.1 and 0.5% and Tween-80 0.1 and 0.5% were tested as buffer B detergents to increase the essential oil solubility in the aqueous reaction mixture. The reaction mixture was incubated for 6 minutes at room temperature (25 °C). Absorbance values were collected after 6 minutes of incubation at 412 nm. The absorbance corresponding to 100% of AChE activity was measured by replacing the EOs/galanthamine solution with 5 μL of pure DMSO. Control and sample blank solutions were prepared by replacing the 150 µL of enzyme solution with 150 µL of buffer B. The percentage of AChE inhibition was measured according to the equation below:

% Inhibition = ΔA (Control) − ΔA (Sample)/ΔA (Control) × 100

ΔA (Control) or (Sample) = A_412_ (Control) or (Sample) − A_412_ (Control Blank) or (Sample Blank)

## 5. Conclusions

The fresh leaves of *Jungia rugosa* Less afforded, by steam distillation, an essential oil in quite a low yield (0.09% by weight). This volatile fraction was composed exclusively of sesquiterpenes, whose major constituents were γ-curcumene (more than 45%) and β-sesquiphellandrene (about 17%). The other two unknown oxygenated sesquiterpenoids were detected among the main constituents (about 7% and 5% of the whole mixture). The EO also manifested a weak inhibition activity against AChE.

## Figures and Tables

**Table 1:** Qualitative and quantitative chemical analyses of the EO from *J. rugosa* fresh leaves.

N.	Compounds	DB-5ms			HP-INNOWax			DB-5ms		HP-INNOWax	
		LRI ^1^	LRI	Ref.	LRI ^1^	LRI	Ref.	(%) ^2^	σ	(%) ^2^	σ
1	α-copaene	1370	1374	[25]	1464	1489	[26]	0.2	0.01	0.3	0.01
2	β-cubebene	1378	1387	[25]	1536	1542	[27]	trace	0.02	trace	-
3	7-epi-sesquithujene	1385	1390	[25]	1576	-		0.9	0.02	trace	-
4	Italicene	1395	1405	[25]	1525	1536	[28]	0.3	0.08	0.5	0.02
5	α-chamipinene	1397	1396	[25]	1552	-		trace	0.05	trace	-
6	undetermined (MW 204)	1412	-		1549	-		1.1	0.02	0.4	0.01
7	α-cis-bergamotene	1420	1411	[25]	1584	1577	[28]	1.6	0.03	1.7	0.05
8	α-trans-bergamotene	1430	1432	[25]	1530	1560	[29]	0.1	0.01	0.3	0.02
9	seychellene	1442	1444	[25]	1663	-		0.1	-	trace	-
10	α-humulene	1448	1452	[25]	1652	1667	[27]	1.1	0.01	0.5	0.01
11	allo-aromadendrene	1451	1458	[25]	1626	1637	[30]	0.1	0.02	trace	-
12	(E)-β-farnesene	1453	1454	[25]	1669	1664	[27]	trace	-	0.8	0.02
13	6-demethoxy ageratochromene	1458	1461	[25]	2083	2075	[31]	0.9	0.02	1.4	0.02
14	2-epi-(E)-caryophyllene	1466	1465	[32]	1674	1669	[32]	0.1	0.01	trace	-
15	ishwarane	1471	1465	[25]	1609	1636	[33]	0.1	0.07	trace	-
16	γ-curcumene	1476	1481	[25]	1685	1692	[27]	47.1	0.70	49.7	0.40
17	ar-curcumene	1479	1479	[25]	1768	1774	[27]	3.4	0.34	4.2	0.32
18	γ-muurolene	1486	1478	[25]	1692	1690	[27]	0.1	0.07	0.8	0.02
19	β-selinene	1489	1489	[25]	1710	1717	[27]	0.3	0.04	1.3	0.02
20	α-zingiberene	1491	1493	[25]	1698	1713	[34]	0.1	0.11	trace	-
21	epi-cubebol	1493	1493	[25]	1943	1928	[35]	1.0	0.13	trace	-
22	β-bisabolene	1505	1505	[25]	1719	1728	[27]	0.9	0.02	1.0	0.02
23	α-cuprenene	1506	1505	[25]	1733	1759	[36]	0.5	0.01	0.6	0.01
24	δ-amorphene	1511	1511	[25]	1704	1710	[37]	trace	-	trace	-
25	γ-cadinene	1513	1513	[25]	1744	1763	[27]	0.7	0.01	0.8	0.01
26	β-sesquiphellandrene	1521	1521	[25]	1762	1771	[27]	17.0	0.20	17.9	0.16
27	8,14-cedranoxide	1549	1541	[25]	1842	1858	[38]	0.1	-	trace	-
28	cis-muurol-5-en-4-α-ol	1569	1559	[25]	2210	2221	[39]	0.1	0.02	trace	-
29	spathulenol	1575	1577	[25]	2141	2140	[40]	0.1	-	0.5	0.01
30	allo-cedrol	1588	1589	[25]	2261	-		0.1	0.03	trace	-
31	sesquithuriferol	1605	1604	[25]	2125	2113	[41]	0.3	0.01	0.3	0.01
32	isolongifolan-7-α-ol	1609	1618	[25]	2117	-		0.3	0.01	0.2	0.11
33	cis-isolongifolanone	1613	1612	[25]	2168	-		0.2	0.01	0.3	0.01
34	undetermined (MW 220)	1627	-		2070	-		6.7	0.10	7.2	0.14
35	3-iso-thujopsanone	1632	1641	[25]	2106	-		0.2	0.13	trace	-
36	allo-aromadendrene epoxide	1634	1639	[25]	2096	2095	[42]	trace	-	trace	-
37	epi-α-muurolol	1642	1640	[25]	2194	2186	[27]	0.1	0.01	0.1	0.01
38	3-thujopsanone	1650	1653	[25]	2265	-		0.3	-	0.2	0.01
39	α-cadinol	1653	1652	[25]	2244	2227	[27]	0.2	0.02	trace	-
40	14-hydroxy-9-epi-(E)-caryophyllene	1658	1668	[25]	2110	-		0.1	0.04	trace	-
41	7-epi-α-eudesmol	1664	1662	[25]	2209	2205	[43]	0.2	0.01	trace	-
42	bulnesol	1667	1670	[25]	2204	2200	[44]	0.2	0.01	trace	-
43	8-cedren-13-ol	1685	1688	[25]	2335	2359	[45]	0.4	0.03	trace	-
44	cyperotundone	1690	1695	[25]	2474	-		2.5	0.04	2.5	0.28
45	zizanal	1701	1697	[25]	2450	-		0.6	0.01	1.4	0.07
46	cis-thujopsenal	1705	1708	[25]	2294	-		trace	-	trace	-
47	14-hydroxy-α-humulene	1713	1713	[25]	-	-		0.1	-	trace	-
48	vetiselinenol	1718	1730	[25]	2445	-		0.2	-	trace	-
49	γ-costol	1742	1745	[25]	2337	-		0.3	0.01	trace	-
50	xanthorrhizol	1749	1751	[25]	2657	2674	[42]	0.6	0.02	1.1	0.27
51	cedryl acetate	1776	1767	[25]	2132	2150	[46]	0.2	0.02	0.3	0.01
52	8-cedren-13-ol acetate	1782	1788	[25]	2248	-		0.3	0.09	trace	-
53	undetermined (MW 262)	1789	-		2272	-		4.7	0.18	3.3	0.07
54	8-α-acetoxyelemol	1793	1792	[25]	-	-		trace	-	trace	-
55	undetermined (MW 280)	2029	-		-	-		1.3	0.30	trace	-
56	n-tricosane	2301	2300	[25]	2300	2300	[47]	0.2	0.07	trace	-
monoterpene hydrocarbons								-		-	
oxygenated monoterpenes								-		-	
sesquiterpene hydrocarbons								75.8%		80.8%	
oxygenated sesquiterpenes								22.3%		18.8%	
others								0.2%		trace	
total								98.3%		99.6%	

^1^ Calculated linear retention index (LRI) according to van den Dool and Kratz [48]; ^2^ Trace for % < 0.1.

**Table 2:** Percent inhibition of AChE by *J. rugosa* EO compared to *L. nobilis* EO and galantamine as positive controls.

Sample	Enzymatic Inhibition (%)	σ
Galanthamine 1.0 µg/mL	49.2	5.2
*Laurus nobilis* EO 38 µg/mL	38.8	4.2
*Jungia rugosa* EO 38 µg/mL	25.9	13.9

## Data Availability

Raw data are available from the authors (K.C.).
